# Complete genome sequencing of Dehalococcoides sp. strain UCH007 using a differential reads picking method

**DOI:** 10.1186/s40793-015-0095-9

**Published:** 2015-11-14

**Authors:** Yoshihito Uchino, Takamasa Miura, Akira Hosoyama, Shoko Ohji, Atsushi Yamazoe, Masako Ito, Yoh Takahata, Ken-ichiro Suzuki, Nobuyuki Fujita

**Affiliations:** Biological Resource Center, National Institute of Technology and Evaluation, 2-10-49 Nishihara, Tokyo, 151-0066 Japan; Taisei Corporation, 344-1 Nase, Kanagawa, 245-0051 Japan

**Keywords:** *Dehalococcoides*, Differential reads picking method, Bioremediation, Reductive dechlorination, Dehalorespiring, Chloroethene

## Abstract

A novel *Dehalococcoides* sp. strain UCH007 was isolated from the groundwater polluted with chlorinated ethenes in Japan. This strain is capable of dechlorinating trichloroethene, *cis*-1,2-dichloroethene and vinyl chloride to ethene. *Dehalococcoides* bacteria are hardly cultivable, so genome sequencing has presented a challenge. In this study, we developed a differential reads picking method for mixed genomic DNA obtained from a co-culture, and applied it to the sequencing of strain UCH007. The genome of strain UCH007 consists of a 1,473,548-bp chromosome that encodes 1509 coding sequences including 29 putative reductive dehalogenase genes. Strain UCH007 is the first strain in the Victoria subgroup found to possess the *pceA*, *tceA* and *vcrA* genes.

**Electronic supplementary material:**

The online version of this article (doi:10.1186/s40793-015-0095-9) contains supplementary material, which is available to authorized users.

## Introduction

Chloroethenes such as PCE, TCE *cis*-1,2-DCE and VC in contaminated soil and groundwater can be removed by reductive dechlorination mediated by anaerobic bacteria. Under anaerobic conditions, dehalorespiring bacteria dechlorinate chloroethenes by mediating the step-wise replacement of chlorine with hydrogen resulting in the conversion of PCE to TCE, DCE isomers, VC, and ethene sequentially. Among many dehalorespiring bacterial isolates, only a few strains of the genus *Dehalococcoides* completely convert chloroethenes to nontoxic ethene, hence they are indispensable for successful bioremediation applications [1–10]. The RDases are essential enzymes for the dehalorespiring activities of *Dehalococcoides* ssp., however, the constitution of RDase genes in each strain varies significantly, resulting in varied dechlorination activities among strains. Among the RDase genes, *vcrA* and *bvcA*, which dechlorinate VC to ethene are essential for complete dechlorination.

In our previous report, we constructed a chloroethene-dechlorinating microbial consortium derived from chloroethene-polluted groundwater in Japan, and identified some operational taxonomic units that were assigned to *Dehalococcoides* by amplicon sequencing of 16S rRNA genes [11]. In this report, we describe a *Dehalococcoides* bacterium designated strain UCH007 isolated from the consortium, and present its complete genome sequence. Strain UCH007, the first *Dehalococcoides* strain isolated in Japan, was phylogenetically affiliated with the Victoria subgroup of the *Dehalococcoides*.

## Organism information

### Classification and features

A *cis*-1,2-DCE-to-ethene dechlorinating enrichment culture was obtained from the microbial consortium [11] by sequentially transferring to fresh media amended with acetate plus H_2_-CO_2_ (80 %:20 %, vol/vol) in the headspace and *cis*-1,2-DCE as the electron acceptor. Following repeated transfers to *cis*-1,2-DCE amended media in the presence of ampicillin or 2-bromoethanesulfonate, several series of dilution-to-extinction culturing and several agar shake processes were performed, and strain UCH007 was obtained in pure culture.

**Fig. 1:**
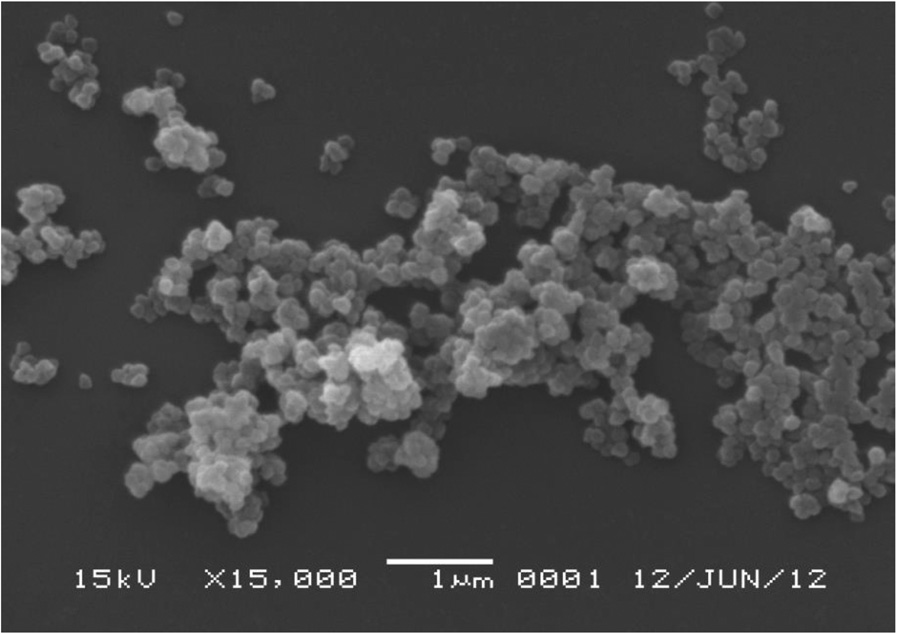
Scanning electron microscopy (SEM) of *Dehalococcoides* sp. strain UCH007. The image was recorded using a JEOL JSM-6060 SEM (JEOL, Tokyo, Japan)

The cells of strain UCH007 were non-motile, non-spore forming and had a disc-shaped morphology with a diameter of 0.1–0.3 μm (Fig. 1). The temperature range for growth of strain UCH007 was between 15 and 35 °C, with optimum growth between 25 and 30 °C. The pH range for growth of strain UCH007 was between 6.2 and 7.7, with an optimum pH between 7.0 and 7.3. The range of NaCl concentrations that allowed for growth of strain UCH007 was 0–1.5 %, with an optimum concentration of 0.3–0.5 %.

Strain UCH007 is a strictly anaerobic bacterium, and its growth depends on the presence of hydrogen as an electron donor, reductive dechlorination substrates such as TCE, *cis*-1,2-DCE, 1,1-DCE and VC as electron acceptors and acetate as a carbon source. Vitamin B_12_ is essential for growth. The strain was observed to accumulate varying amounts of VC during TCE (or *cis*-1,2-DCE)-to-ethene dechlorination, but growth tended to be coupled with the reductive dechlorination of VC.

**Fig. 2:**
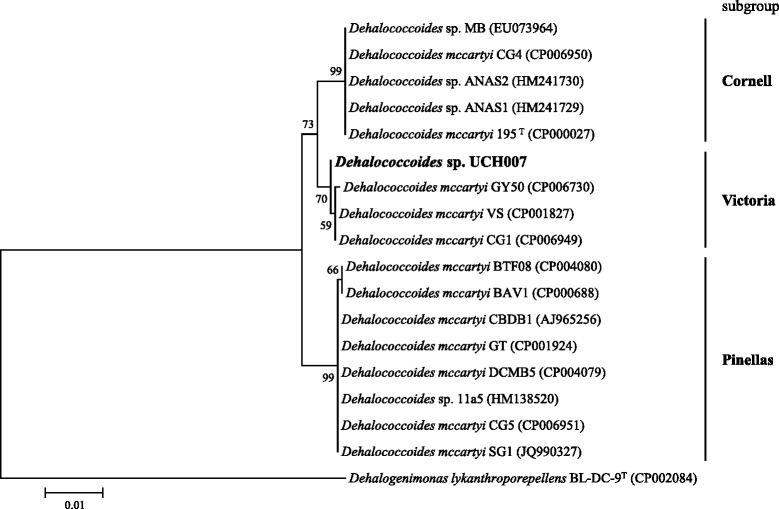
Phylogenetic tree showing the position of *Dehalococcoides* sp. strain UCH007. The tree was constructed using maximum-likelihood estimation with bootstrap values using the MEGA5.2 software [33]. *Dehalogenimonas lykanthroporepellens* BL-DC-9^T^ was used as an outgroup

*Dehalococcoides* strains isolated to date shared more than 98 % 16S rRNA gene sequence similarity with each other, and grouped into three subgroups designated the Pinellas, Victoria and Cornell subgroups [1]. Phylogenetic analysis based on 16S rRNA gene sequences shows that strain UCH007 belonged to the Victoria subgroup, and the most closely related strain was *D. mccartyi* strain VS with 99.92 % similarity (Fig. 2). The most distantly related strain was *D. mccartyi* strain CBDB1 with 98.91 % similarity.

## Genome sequencing information

### Genome project history

Strain UCH007 is the first *Dehalococcoides* isolate from Japan and is one of the few strains found to convert toxic chloroethenes to nontoxic ethene. It was selected for sequencing on the basis of its rarity and importance in bioremediation. Table 1 presents the project information and its association with MIGS version 2.0 compliance [12]. A summary of the project information is shown in Table 2.

**Table 1:** Classification and general features of *Dehalococcoides* sp. strain UCH007 [12]

MIGS ID	Property	Term	Evidence code
	Classification	Domain *Bacteria*	TAS [34]
		Phylum “*Chloroflexi*”	TAS [35, 36]
		Class *Dehalococcoidia*	TAS [1]
		Order *Dehalococcoidales*	TAS [1]
		Family *Dehalococcoidaceae*	TAS [1]
		Genus *Dehalococcoides*	TAS [1]
		Species *Dehalococcoides* sp.	TAS [1]
		Strain UCH007 (Taxonomy ID: 1522671)	
	Gram stain	Gram-indifferent	TAS [1]
	Cell shape	Disk shape	IDA
	Motility	Non-motile	IDA
	Sporulation	Non sporulation	IDA
	Temperature range	15–35 °C	IDA
	Optimum temperature	25–30 °C	IDA
	pH range; Optimum	6.2–7.7; 7.0–7.3	IDA
	Carbon source	Acetate	IDA
MIGS-6	Habitat	Groundwater	IDA
MIGS-6.3	Salinity	0–1.5 % NaCl (w/v)	IDA
MIGS-22	Oxygen requirement	Anaerobic	IDA
MIGS-15	Biotic relationship	Free living	TAS [1]
MIGS-14	Pathogenicity	None	NAS
MIGS-4	Geographic location	Japan	IDA
MIGS-5	Sample collection	2009	IDA
MIGS-4.1	Latitude	undisclosed	IDA
MIGS-4.2	Longitude	undisclosed	IDA
MIGS-4.4	Altitude	−2.5 to −11.0 m	IDA

Evidence codes - *IDA* inferred from direct assay, *TAS* traceable author statement (i.e., a direct report exists in the literature), *NAS* non-traceable author statement (i.e., not directly observed for the living, isolated sample, but based on a generally accepted property for the species, or anecdotal evidence). These evidence codes are from the Gene Ontology project [37]

**Table 2:** Project information

MIGS ID	Property	Term
MIGS 31	Finishing quality	Finished
MIGS-28	Libraries used	454 standard library and Illumina MiSeq library (paired-end)
MIGS 29	Sequencing platforms	454 GS FLX Titanium, Illumina MiSeq
MIGS 31.2	Fold coverage	20.25× 454 GS FLX Titanium
		98.35× Illumina MiSeq
MIGS 30	Assemblers	Newbler 2.6
MIGS 32	Gene calling method	MiGAP
	Genome database release	DDBJ
	Locus Tag	UCH007
	Genbank ID	AP014722
	Genbank Date of Release	February 15, 2015
	BIOPROJECT	PRJDB2892
MIGS 13	Source Material Identifier	UCH007
	Project relevance	The microbial biodegradation of pollutants is attracting attention to find feasible ways to clean-up contaminated environments.

### Growth conditions and genomic DNA preparation

Strain UCH007 was pure-cultured in 300 mL of bicarbonate-buffered medium supplemented with 10 μM of *cis*-1,2-DCE for 47 days [3], however, the number of cells was insufficient for genome sequencing using next-generation sequencers. So, WGA using the pure culture as a template was performed using the REPLI-g Mini Kit (Qiagen GmbH, Hilden, Germany) according to the manufacturer’s instructions.

Strain UCH007 was also co-cultured with *Sulfurospirillum cavolei* UCH003 [13] in bicarbonate-buffered medium for 36 days. Cells were harvested from 100 mL of the culture by centrifugation (12,000 × *g*, 15 min, 4 °C). Total DNA was extracted using the DNeasy Blood and Tissue Kit (Qiagen) according to the manufacturer’s instructions. The effects of strain UCH003 on the growth of strain UCH007, will be described in a separate report (manuscript in preparation).

### Genome sequencing and assembly

**Fig. 3:**
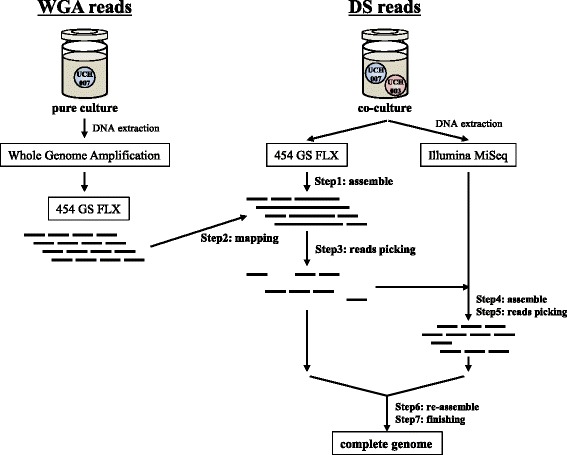
The scheme of the differential reads picking method for sequencing of *Dehalococcoides* sp. strain UCH007

It was difficult to obtain sufficient genomic DNA for direct shotgun sequencing from the pure culture of strain UCH007. It was also difficult to construct a complete genome sequence using reads generated by WGA because of the high abundance of chimeric reads. Therefore, direct shotgun sequencing was performed using the mixed genomic DNA obtained from the co-culture. Then, the differential reads picking method (Fig. 3) was applied to pick up reads that originated from strain UCH007.

The DNA obtained by WGA was sequenced using a 454 GS FLX Titanium pyrosequencer (Roche, Basel, Switzerland), and generated 85,621 reads (WGA reads). The mixed genomic DNA extracted from the co-culture was directly sequenced using 454 GS FLX and Illumina MiSeq sequencers (Illumina, San Diego, CA, USA), and generated 213,427 reads and 3,332,948 reads with 251 bp paired-end sequencing, respectively (DS reads). The reads from the MiSeq were trimmed using sickle software with default parameters [14].

After assembling the DS reads from the 454 GS FLX using Newbler 2.6 (Roche) (Fig. 3; Step 1), the WGA reads were mapped to the resulting contigs using Newbler 2.8 (Fig. 3; Step 2). The DS reads from the 454 GS FLX that were contained in the mapped contigs were recovered, these were considered to originate from strain UCH007, yielding 47,262 reads (29,841,879 bp) (Fig. 3; Step 3). Next, these reads and 2.5 million paired-end reads and 8,414 single-end reads from the MiSeq (approximately 100 × coverage against the *D. mccartyi* VS genome) were assembled using Newbler 2.6 software (Fig. 3; Step 4). Then the MiSeq reads co-assembled with the 454 GS FLX reads were picked, yielding 620,022 paired-end reads and 1,874 single-end reads (144,540,399 bp and 383,354 bp, respectively) (Fig. 3; Step 5). Finally, the picked DS reads both from 454 GS FLX and MiSeq were re-assembled, yielding 13 contigs (Fig. 3; Step 6). Genome closure was accomplished by manual adjustment of the assembly (Fig. 3; Step 7).

### Genome annotation

The complete sequence of the chromosome was analyzed using MiGAP [15], which uses MetaGeneAnnotator [16] for predicting protein-coding genes, tRNAscan-SE [17] for tRNA genes and RNAmmer [18] for rRNA genes. The functions of the predicted protein-coding genes were assigned based on information in the Uniprot [19], Interpro [20], HAMAP [21] and KEGG [22] databases, and an in-house database composed of manually curated microbial genome sequences, as reported previously [23]. Genes in internal clusters were detected using BLASTclust with thresholds of 70 % covered length and 30 % sequence identity [24]. Signal peptides and transmembrane helices were predicted using SignalP [25] and TMHMM [26], respectively.

## Genome properties

**Fig. 4:**
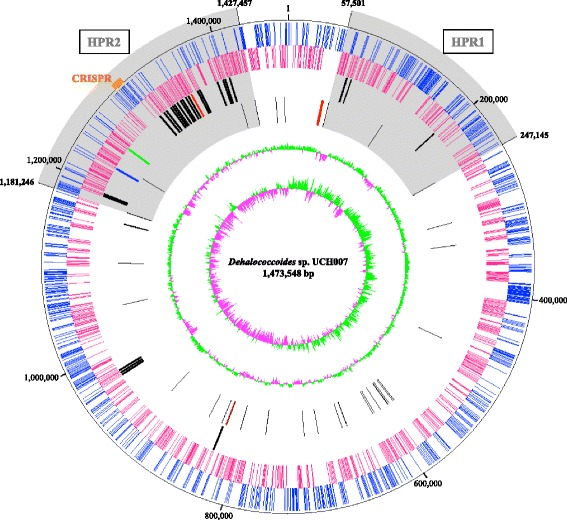
Graphical circular map of the genome of *Dehalococcoides* sp. strain UCH007. The map was drawn using ArcWithColor [38]. From outside to the center: genes on the forward strand, genes on the reverse strand, *rdhA* genes (*pceA* gene, *red*; *tceA* gene, *blue*; *vcrA* gene, *green*), RNA genes (rRNAs, *red*; tRNAs, *black*), GC content, GC skew

The genome of strain UCH007 consisted of a circular chromosome of 1,473,548 bp with a 46.91 % G+C content. The chromosome was predicted to contain 1,509 protein coding genes, 47 tRNA genes and 3 rRNA genes (Table 3 and Fig. 4). The distribution of protein coding genes into COG functional categories is shown in Table 4.

**Table 3:** Genome statistics

Attribute	Value	% of total
Genome size (bp)	1,473,548	100.00
DNA coding (bp)	1,323,945	89.85
DNA G+C (bp)	691,289	46.91
DNA scaffolds	1	
Total genes	1,559	100.00
Protein coding genes	1,509	96.79
RNA genes	50	3.21
Pseudo genes	2	0.13
Genes in internal clusters	311	19.95
Genes with function prediction	1,006	64.53
Genes assigned to COGs	1,150	73.77
Genes with Pfam domains	1,224	78.51
Genes with signal peptides	129	8.27
Genes with transmembrane helices	345	22.13
CRISPR repeats	1

**Table 4:** Number of genes associated with general COG functional categories

Code	Value	% of total	Description
J	132	8.75	Translation
A	0	0.00	RNA processing and modification
K	100	6.63	Transcription
L	92	6.10	Replication, recombination and repair
B	2	0.13	Chromatin structure and dynamics
D	14	0.93	Cell cycle control, mitosis and meiosis
V	18	1.19	Defense mechanisms
T	56	3.71	Signal transduction mechanisms
M	34	2.25	Cell wall/membrane biogenesis
N	10	0.66	Cell motility
U	28	1.86	Intracellular trafficking and secretion
O	54	3.58	Posttranslational modification, protein turnover, chaperones
C	136	9.01	Energy production and conversion
G	41	2.72	Carbohydrate transport and metabolism
E	118	7.82	Amino acid transport and metabolism
F	49	3.25	Nucleotide transport and metabolism
H	70	4.64	Coenzyme transport and metabolism
I	28	1.86	Lipid transport and metabolism
P	57	3.78	Inorganic ion transport and metabolism
Q	10	0.66	Secondary metabolites biosynthesis, transport and catabolism
R	131	8.68	General function prediction only
S	98	6.49	Function unknown
–	359	23.79	Not in COGs

## Insights from the genome sequence

The ANI is becoming widely accepted as a method to delineate bacterial species, with 95–96 % ANI value corresponding to 70 % DNA relatedness [27, 28]. Löffler et al. noted that strains BAV1, CBDB1 and GT (Pinellas subgroup) showed lower ANI values, 86–87 %, to strain VS (Victoria subgroup) and strain 195 (Cornell subgroup) [1]. However, they proposed only one species, *D. mccartyi*, to accommodate all six isolates belonging to three different subgroups because of the high similarity of gene contents, and morphological and physiological characteristics. We recalculated ANI values, based on ANIb using the JSpecies program with default settings, to make full use of the accumulating genomic sequences of *Dehalococcoides*. The results showed that strain UCH007 was closely related to strains GY50, CG1 and VS (Victoria subgroup) with 98.52, 97.99 and 97.07 % ANI values, respectively (Additional file 1: Table S1), which were above the species threshold [27]. By comparison, the strain UCH007 and other members of Victoria subgroup were more distantly related to strains 195^T^ and CG4 (Cornell subgroup) with ANI values of 89.20–89.40 %, and other strains (Pinellas subgroup) with ANI values of 85.95–86.96 %. In addition, the ANI values between the strain 195^T^ or CG4, and strains in the Pinellas subgroup were 85.22–86.02 %. Altogether, all strains in each of three subgroups, each subgroup consisting of at least two strains, showed ANI values lower than the 95–96 % threshold to all strains in other two subgroups (Additional file 1: Table S1). These results suggest that three subgroups of *Dehalococcoides* are to be considered three separate species [27].

The genome of strain UCH007 harbors 29 *rdhA* and *rdhB* gene clusters, and four of these 29 RdhA proteins (UCH007_00760, UCH007_09900, UCH007_09930 and UCH007_13640) showed low similarities (<55 %) to those in other strains. HPRs have been designated on the genomes of strains within the genus *Dehalococcoides* [9, 29, 30], and three and 22 *rdhA* genes in strain UCH007 locate in HPR1 and HPR2, respectively (Fig. 4). Strain BTF08, belonging to the Pinellas subgroup, was the first strain reported to contain the *pceA*, *tceA* and *vcrA* genes, encoding key enzymes in the reductive dechlorination of chloroethenes [9]. Strain UCH007 also contains orthologues of *pceA* (UCH007_13880), *tceA* (UCH007_12670) and *vcrA* (UCH007_12960), and is the first example of a strain containing these genes in the Victoria subgroup (Additional file 2: Table S2). The *vcrA* gene of strain UCH007 was detected in a genomic island located downstream of the *ssrA* gene as is the case with other *Dehalococcoides* strains [9, 31].

Clustered Regularly Interspaced Short Palindromic Repeat (CRISPR)-associated genes are detected on the HPR2 in the genome of strain UCH007 (UCH007_13260-13330), and 40 spacer regions (start position: 1,300,493 bp, end position: 1,302,966 bp) are predicted using the CRISPRfinder program online [32]. CRISPR-associated genes have only ever been found in the Pinellas subgroup, and strains CBDB1, DCMB5 and GT [9, 29, 30], so this is the first report of a CRISPR region in the Victoria subgroup. A bi-directional BLASTP search of the CRISPR-associated proteins showed sequence identity of more than 73 % between strain UCH007 and other strains (Additional file 3: Table S3). The direct repeat was 29 bp in length, and the consensus sequence (5′-GTATTCCCCACGCgTGTGGGGGTGAACCG-3′) was conserved among the four strains, with the exception of the base shown in lowercase [32]. Therefore, these CRISPRs seem to share a common evolutionary origin.

## Conclusions

Here we reported the isolation and complete genome sequence of *Dehalococcoides* strain UCH007, which can dechlorinate chloroethenes to ethene. The genome sequence showed that the strain UCH007 is the first strain in the Victoria subgroup of *Dehalococcoides* revealed to possess *pceA*, *tceA* and *vcrA* genes on the chromosome. As this strain is currently considered to be used in the bioaugmentation of chloroethenes-contaminated groundwater, this information will be useful for monitoring and improve the bioaugmentation process through, for example, metagenomic and metatranscriptomic analyses.

## Additional files

Additional file 1: Table S1. Phylogenetic analysis using whole genome sequences and 16S rRNA genes. (XLSX 12 kb) Additional file 2: Table S2. Comparison of reductive dehalogenases of strain UCH007 with those of other strains. (XLSX 12 kb) Additional file 3: Table S3. Comparison of CRISPR-associated proteins of strain UCH007 with those of other strains. (XLSX 10 kb)

## Abbreviations

ANI: average nucleotide identities
ANIb: average nucleotide identities by BLAST
*cis*-1,2-DCE: *cis*-1,2-dichloroethene
HPR: high plasticity region
MiGAP: microbial genome annotation pipeline
PCE: tetrachloroethene
RDase: reductive dehalogenase
TCE: trichloroethene
VC: vinyl chloride
WGA: whole genome amplification

## References

[1] Löffler FE, Yan J, Ritalahti KM, Adrian L, Edwards EA, Konstantinidis KT (2013). *Dehalococcoides mccartyi* gen. nov., sp. nov., obligately organohalide-respiring anaerobic bacteria relevant to halogen cycling and bioremediation, belong to a novel bacterial class, *Dehalococcoidia* classis nov., order *Dehalococcoidales* ord. nov. and family *Dehalococcoidaceae* fam. nov., within the phylum *Chloroflexi*. Int J Syst Evol Microbiol.

[2] Maymó-Gatell X, Chien Y, Gossett JM, Zinder SH (1997). Isolation of a bacterium that reductively dechlorinates tetrachloroethene to ethene. Science.

[3] He J, Ritalahti KM, Yang KL, Koenigsberg SS, Löffler FE (2003). Detoxification of vinyl chloride to ethene coupled to growth of an anaerobic bacterium. Nature.

[4] He J, Sung Y, Krajmalnik-Brown R, Ritalahti KM, Löffler FE (2005). Isolation and characterization of *Dehalococcoides* sp. strain FL2, a trichloroethene (TCE)- and 1,2-dichloroethene-respiring anaerobe. Environ Microbiol.

[5] Sung Y, Ritalahti KM, Apkarian RP, Löffler FE (2006). Quantitative PCR confirms purity of strain GT, a novel trichloroethene-to-ethene-respiring *Dehalococcoides* isolate. Appl Environ Microbiol.

[6] Müller JA, Rosner BM, Von Abendroth G, Meshulam-Simon G, McCarty PL, Spormann AM (2004). Molecular identification of the catabolic vinyl chloride reductase from *Dehalococcoides* sp. strain VS and its environmental distribution. Appl Environ Microbiol.

[7] Cheng D, He J (2009). Isolation and characterization of "*Dehalococcoides*" sp. strain MB, which dechlorinates tetrachloroethene to trans-1,2-dichloroethene. Appl Environ Microbiol.

[8] Lee PK, Cheng D, Hu P, West KA, Dick GJ, Brodie EL (2011). Comparative genomics of two newly isolated *Dehalococcoides* strains and an enrichment using a genus microarray. ISME J.

[9] Pöritz M, Goris T, Wubet T, Tarkka MT, Buscot F, Nijenhuis I (2013). Genome sequences of two dehalogenation specialists – *Dehalococcoides mccartyi* strains BTF08 and DCMB5 enriched from the highly polluted Bitterfeld region. FEMS Microbiol Lett.

[10] Wang S, Chng KR, Wilm A, Zhao S, Yang KL, Nagarajan N (2014). Genomic characterization of three unique *Dehalococcoides* that respire on persistent polychlorinated biphenyls. Proc Natl Acad Sci USA.

[11] Miura T, Yamazoe A, Ito M, Ohji S, Hosoyama A, Takahata Y (2015). The impact of injections of different nutrients on bacterial community and its dechlorination activity in chloroethene-contaminated groundwater. Microbes Environ.

[12] Field D, Garrity G, Gray T, Morrison N, Selengut J, Sterk P (2008). The minimum information about a genome sequence (MIGS) specification. Nat Biotechnol..

[13] Miura T, Uchino Y, Tsuchikane K, Ohtsubo Y, Ohji S, Hosoyama A (2015). Complete genome sequence of Sulfurospirillum strain UCH001 and UCH003 isolated from groundwater in Japan. Genome Announc..

[14] najoshi/sickle [https://github.com/najoshi/sickle]. Access date 6/11/2015.

[15] Microbial Genome Annotation Pipeline [http://www.migap.org/index.php/en]. Access date 6/11/2015.

[16] Noguchi H, Taniguchi T, Itoh T (2008). MetaGeneAnnotator: detecting species-specific patterns of ribosomal binding site for precise gene prediction in anonymous prokaryotic and phage genomes. DNA Res..

[17] Lowe TM, Eddy SR (1997). tRNAscan-SE: a program for improved detection of transfer RNA genes in genomic sequence. Nucleic Acids Res..

[18] Lagesen K, Hallin P, Rødland EA, Staerfeldt HH, Rognes T, Ussery DW (2007). RNAmmer: consistent and rapid annotation of ribosomal RNA genes. Nucleic Acids Res..

[19] UniProt Consortium (2010). The universal protein resource (UniProt) in 2010. Nucleic Acids Res.

[20] Mulder NJ, Apweiler R, Attwood TK, Bairoch A, Bateman A, Binns D (2007). New developments in the InterPro database. Nucleic Acids Res..

[21] Lima T, Auchincloss AH, Coudert E, Keller G, Michoud K, Rivoire C (2009). HAMAP: a database of completely sequenced microbial proteome sets and manually curated microbial protein families in UniProtKB/Swiss-Prot. Nucleic Acids Res..

[22] Kanehisa M, Araki M, Goto S, Hattori M, Hirakawa M, Itoh M (2007). KEGG for linking genomes to life and the environment. Nucleic Acids Res..

[23] Shintani M, Hosoyama A, Ohji S, Tsuchikane K, Takarada H, Yamazoe A (2013). Complete genome sequence of the carbazole degrader Pseudomonas resinovorans strain CA10 (NBRC 106553). Genome Announc..

[24] BLASTclust [http://toolkit.tuebingen.mpg.de/blastclust]. Access date 6/11/2015.

[25] SignalP [http://www.cbs.dtu.dk/services/SignalP/]. Access date 6/11/2015.

[26] TMHMM. Transmembrane domain prediction. [http://www.cbs.dtu.dk/services/TMHMM/]. Access date 6/11/2015.

[27] Richter M, Rosselló-Móra R (2009). Shifting the genomic gold standard for the prokaryotic species definition. Proc Natl Acad Sci U S A..

[28] Goris J, Konstantinidis KT, Klappenbach JA, Coenye T, Vandamme P, Tiedje JM (2007). DNA-DNA hybridization values and their relationship to whole-genome sequence similarities. Int J Syst Evol Microbiol..

[29] McMurdie PJ, Behrens SF, Müller JA, Göke J, Ritalahti KM, Wagner R (2009). Localized plasticity in the streamlined genomes of vinyl chloride respiring Dehalococcoides. PLoS Genet..

[30] Kube M, Beck A, Zinder SH, Kuhl H, Reinhardt R, Adrian L (2005). Genome sequence of the chlorinated compound-respiring bacterium Dehalococcoides species strain CBDB1. Nat Biotechnol..

[31] McMurdie PJ, Hug LA, Edwards EA, Holmes S, Spormann AM (2011). Site-specific mobilization of vinyl chloride respiration islands by a mechanism common in Dehalococcoides. BMC Genomics..

[32] UUU CRISPRfinder program online [http://crispr.u-psud.fr/Server/]. Access date 6/11/2015.

[33] Tamura K, Peterson D, Peterson N, Stecher G, Nei M, Kumar S (2011). MEGA5: molecular evolutionary genetics analysis using maximum likelihood, evolutionary distance, and maximum parsimony methods. Mol Biol Evol..

[34] Woese CR, Kandler O, Wheelis ML (1990). Towards a natural system of organisms: proposal for the domains Archaea, Bacteria, and Eucarya. Proc Natl Acad Sci U S A..

[35] Castenholz RW, Boone DR, Castenholz RW, Garrity GM (2001). Class I: “Chloroflexi”. Bergey’s Manual of Systematic Bacteriology.

[36] Oren A, Garrity GM (2013). List of new names and new combinations previously effectively, but not validly, published. Int J Syst Evol Microbiol..

[37] Ashburner M, Ball CA, Blake JA, Botstein D, Butler H, Cherry JM (2000). Gene ontology: tool for the unification of biology. The Gene Ontology Consortium. Nat Genet..

[38] Ohtsubo Y, Ikeda-Ohtsubo W, Nagata Y, Tsuda M (2008). GenomeMatcher: a graphical user interface for DNA sequence comparison. BMC Bioinformatics..

